# Involvement of membrane palmitoylated protein 6 (MPP6) in synapses of mouse cerebrum

**DOI:** 10.1007/s00418-025-02378-1

**Published:** 2025-05-13

**Authors:** Yurika Saitoh, Sayaka Motofuji, Akio Kamijo, Tatsuo Suzuki, Takahiro Yoshizawa, Takeharu Sakamoto, Kiyokazu Kametani, Nobuo Terada

**Affiliations:** 1 Center for Medical Education, Teikyo University of Science, 2-2-1 Senjusakuragi, Adachi-Ku, Tokyo, 120-0045 Japan; 2 Division of Biosciences, Teikyo University of Science Graduate School of Science & Engineering, Adachi-ku, Tokyo, Japan; 3 Division of Basic & Clinical Medicine, Nagano College of Nursing, Komagane City, Nagano, Japan; 4 Department of Molecular and Cellular Physiology, Shinshu University School of Medicine, Matsumoto City, Nagano, Japan; 5 Division of Animal Research, Research Center for Advanced Science and Technology, Shinshu University, Matsumoto City, Nagano, Japan; 6 Department of Cancer Biology, Institute of Biomedical Science, Kansai Medical University, Hirakata City, Osaka, Japan; 7 Health Science Division, Department of Medical Sciences, Shinshu University Graduate School of Medicine, Science and Technology, 3-1-1 Asahi, Matsumoto City, Nagano, 390-8621 Japan

**Keywords:** MPP6, Membrane skeletal protein, Cerebrum, MPP families

## Abstract

Membrane palmitoylated protein 6 (MPP6), a membrane skeletal protein, is expressed not only in the peripheral nervous system (PNS) but also in the central nervous system (CNS). In this study, we investigated the localization of MPP6 and its associated protein complexes in the mouse cerebrum, as well as its effects on behavior using MPP6 protein-deficient (*Mpp6* −/−) mice. MPP6 was detected in mouse cerebral lysates and synaptic membrane fractions, where it formed protein complexes with other MPP family members, including MPP1, MPP2, and calcium/calmodulin-dependent serine protein kinase (CASK). However, the amounts of these complexes did not differ between *Mpp6* −/− and wild-type (*Mpp6* +/+) mice. Immunohistochemistry revealed that MPP6 was localized at synapses throughout the cerebrum, particularly in the postsynaptic regions. Ultrastructural analysis showed that synaptic cleft distances and postsynaptic density thickness were slightly reduced in *Mpp6* −/− mice compared with *Mpp6* +/+ mice. In the elevated plus-maze test, a *Mpp6* −/− mouse exhibited unusual behavior not observed in *Mpp6* +/+ mice, although there was no statistically significant difference in the time spent in the open and closed arms between the two groups. Locomotor activity measurements revealed that MPP6 −/− mice were more active at midnight and less active from morning to noon than *Mpp6* +/+ mice, implying alterations in sleep–wake regulation. These findings suggest that MPP6 plays a role in synaptic function by forming protein complexes with other MPP family members and signaling proteins.

## Introduction

Membrane palmitoylated protein 6 (MPP6) is one of the seven members of the MPP family (MPP1–MPP7). The MPP family proteins contain six domains: two consecutive L27 (Lin2/Lin7) domains (except for MPP1), the postsynaptic density protein 95 (PSD95)/*Drosophila* discs large tumor suppressor (Dlg)/zonula occludens 1 (ZO1) [PDZ] domain, the Src-homology 3 (SH3) domain, the HOOK domain, and a catalytically inactive guanylate kinase-like (GK) domain (Chytla et al. 2020). These domains facilitate interactions with various intramembrane proteins, such as adhesion molecules, and with membrane skeletal proteins, including protein 4.1 family members and actin (Chytla et al. 2020). We previously reported that in the peripheral nervous system (PNS), MPP6 forms a protein complex with 4.1G and cell adhesion molecule 4 (CADM4) (Saitoh et al. 2019, 2017; Terada et al. 2023), which is analogous to the 4.1R–MPP1–glycophorin C membrane skeletal protein complex found in erythrocytes (Narla and Mohandas 2017). In Schwann cells of the PNS, 4.1G transports MPP6 and CADM4, while MPP6 transports Lin7, contributing to myelin formation (Saitoh et al. 2019).

In contrast, the expression, localization, and associated proteins of MPP6 in the central nervous system (CNS) remain poorly understood. Notably, recent genome-wide association studies (GWAS) have suggested that *MPP6* may be associated with human mental disorders such as schizophrenia (Ganapathiraju et al. 2016; Greenwood et al. 2019; Lin et al. 2016; Ripke et al. 2014). In addition, *MPP6* has been identified as a novel gene involved in sleep regulation within sleep center neurons (Khoury et al. 2021). Therefore, in this study, we investigated the localization of MPP6 and its protein complexes in the mouse cerebrum, as well as its potential role in behavior, using MPP6-deficient mice.

## Materials and methods

### *Mpp6* gene-mutated mice

All animal experiments were performed in accordance with the guidelines of the Animal Care and Use Committee of Shinshu University. *Mpp6* gene-mutated mice were generated using CRISPR-Cas9 genome editing, targeting the amino acid sequence just before the PDZ domain of the MPP6 protein, as previously reported (Saitoh et al. 2019).

### Preparation of cerebral lysates and synaptic plasma membrane fractions, and western blot analysis

The cerebrum of 3- to 4-month-old *Mpp6* +/+ or *Mpp6* −/− mice (three mice per group) were lysed in TENT buffer [20 mM Tris (hydroxymethyl) aminomethane (pH 7.4), 1 mM ethylenediaminetetraacetic acid (EDTA), 50 mM NaCl, 1% Triton X-100] containing a protease inhibitor cocktail (Sigma, St. Louis, MI, USA). The lysates were centrifuged at 15,000 × *g* at 4 °C for 15 min, and the protein concentration was adjusted using a protein assay kit (ThermoFisher, Rockford, IL, USA). Synaptic plasma membrane fractions were prepared from mice and Wistar rats (male, 6 weeks old) as described previously (Suzuki et al. 2018).

The samples were mixed with Laemmli sample buffer containing β-mercaptoethanol (β-ME) and subjected to sodium dodecyl sulfate–polyacrylamide gel electrophoresis (SDS-PAGE) on a 5–20% gradient gel, and transferred onto membranes. The membranes were probed with the following primary antibodies (Abs): rabbit polyclonal anti-MPP6 Abs targeting different regions [N-terminal region (MPP6 Ab no. 1: GeneTex; no. GTX108010), second L27 domain (MPP6 Ab no. 2: ATLAS (Stockholm, Sweden); no. HPA019085), central region (MPP6 Ab no. 3: GeneTex; no. GTX117969), C-terminal region (MPP6 Ab no. 4: originally produced by Saitoh et al. (2019)], rabbit polyclonal anti-MPP1 Ab (originally produced by Zhang et al. (2005), rabbit polyclonal anti-MPP2 Ab [GeneTex; no. GTX103908 (Yamada et al. 2022)], rabbit polyclonal anti-Lin7 Ab (GeneTex; no. GTX117114), mouse monoclonal anti-CASK Ab (NeuroMab, Davis, CA, USA; no. K56A/50), mouse monoclonal anti-actin Ab (Wako, Osaka, Japan; clone no. 2F3). After incubation with horseradish peroxidase (HRP)-conjugated anti-rabbit or anti-mouse immunoglobulin G (IgG) secondary Ab (ThermoFisher), the membranes were treated with a chemiluminescent detection solution (ThermoFisher) and visualized using a detection system (LAS4000: FUJIFILM, Tokyo, Japan).

Band intensities were quantified using Photoshop software (Adobe, San Jose, CA, USA), and the resulting values were analyzed using the following steps. First, actin protein levels in *Mpp6* +/+ and *Mpp6 −/− *mice (three mice per group) were compared to confirm that there was no significant difference between the groups. Next, the levels of MPP1, MPP2, Lin7, and CASK proteins were normalized to actin protein levels in the three *Mpp6* +/+ and three *Mpp6* −/− mice. Finally, the relative expression levels of MPP1, MPP2, Lin7, and CASK proteins in *Mpp6* −/− samples were determined by setting the corresponding *Mpp6* +/+ sample values to 1.0. Statistical analysis was performed using the Mann–Whitney *U* test.

### Immunoprecipitation study

Immunoprecipitation analyses were performed to examine the interactions between MPP6 and associated proteins in the mouse cerebrum. Cerebral lysates were prepared using TENT buffer containing a protease inhibitor cocktail, followed by centrifugation at 12,000 × *g* at 4 °C for 10 min. The lysates were pretreated with protein G-sepharose (GE Healthcare, Piscataway, NJ, USA) at 4 °C for 1 h to remove nonspecific proteins, including intrinsic mouse IgGs. The samples were then incubated with rabbit polyclonal anti-MPP6 Ab no. 2 at 4 °C for 2 h. Immunoprecipitated proteins were collected using protein G-sepharose at 4 °C for 1 h, eluted by boiling in Laemmli sample buffer containing β-ME, and subjected to SDS-PAGE on a 5–20% gradient gel and western blot analyses using Abs against MPP1, MPP2, or CASK, followed by a HRP-conjugated anti-rabbit or mouse IgG Ab. Blots were visualized using a chemiluminescent detection system.

### Light microscopic immunohistochemistry

Anesthetized 3- to 4-month-old C57BL/6J mice were perfused via the heart with 2% paraformaldehyde in 0.1 M phosphate buffer (PB, pH 7.4). The cerebrum was dissected and immersed in the same fixative at 4 °C for 2 h, embedded in 30% sucrose, and sectioned into floating sections (20 μm) using a cryostat. Coronal sections corresponding to Allen Brain Atlas images 55–70 were obtained and subjected to antigen retrieval by microwave heating in ImmunoSaver™ (FUJIFILM Wako, Osaka, Japan) (Namimatsu et al. 2005). Sections were incubated with rabbit anti-MPP6 Ab no. 2, followed by biotinylated anti-rabbit IgG Ab (Vector Lab, Burlingame, CA, USA) at room temperature for 2 h. After treatment with a HRP-conjugated avidin–biotin complex (ThermoFisher) for 30 min, sections were visualized using metal-enhanced diaminobenzidine (ThermoFisher), sections were treated with 0.04% OsO_4_ for 60 s, and mounted with Vectashield™. They were observed under a light microscope (BX51: Olympus, Tokyo, Japan) using objective lenses of 4, 10, 20, and 40×. Images were captured with a digital camera (FX630: Flovel, Tokyo, Japan) at a resolution of 300 dpi.

For double-fluorescence immunostaining, some sections were incubated overnight at 4 °C with rabbit polyclonal anti-MPP6 Ab no. 2 and mouse monoclonal anti-GluN1 (BioLegend, San Diego, CA, USA; no. MMS-5145). The sections were then treated with Alexa Fluor 594-conjugated anti-rabbit IgG (Invitrogen) and Alexa Fluor 488-conjugated anti-mouse IgG (Cell Signaling Technology, Danvers, MA, USA) at room temperature for 2 h. After mounting with Vectashield™, the cerebral cortex regions were examined using a confocal laser-scanning microscope (LSM880; Zeiss, Oberkochen, Germany) with a 40× objective lens. All light microscopic images were arranged using Photoshop (ver. 26.5) at a resolution of 300 dpi.

### Post-embedding immunoelectron microscopy

Anesthetized 3- to 4-month-old C57BL/6 J mice were perfused via the heart with 2% paraformaldehyde and 0.25% glutaraldehyde in 0.1 M PB and post-fixed at 4 °C for 1 d. Cerebral cortex samples (Allen Brain Atlas images 60–70) from the right side above the corpus callosum were dissected, dehydrated in graded ethanol, and embedded in LR-Gold resin (Polysciences Inc., Warrington, PA, USA) under ultraviolet irradiation at −20 °C for 2 d. Ultrathin sections from cortical layers II/III–V were mounted on Formvar-coated grids, subjected to antigen retrieval by heating in ImmunoSaver™ at 95 °C for 10 min, and incubated with rabbit anti-MPP6 Ab no. 2, followed by 18 nm gold-conjugated anti-rabbit IgG Ab (Jackson ImmunoResearch, West Grove, PA, USA). Sections were observed using a transmission electron microscope (JEM-1400: JEOL, Tokyo, Japan).

### Electron microscopy for ultrastructural analysis

Perfusion-fixed 4-month-old male *Mpp6* +/+ and *Mpp6* −/− mice (three per group) were analyzed. The cerebral cortex (Allen Brain Atlas images 60–70) was dissected, post-fixed in 1% OsO_4_ in 0.1 M PB for 2 h, dehydrated, and embedded in epoxy resin. Ultrathin sections were obtained from cortical layers II/III–V, stained with uranyl acetate and lead citrate, and examined using a transmission electron microscope. The width of synaptic clefts and thickness of the postsynaptic density (PSD) were measured in type I synapses from *Mpp6* +/+ (*n* = 280) and *Mpp6* −/− (*n* = 375) mice. All electron microscopic images were analyzed using Photoshop (ver. 26.5) at a resolution of 300 dpi. Statistical analyses of synaptic cleft width and PSD thickness in *Mpp6* +/+ and *Mpp6* −/− mice (three per group) were performed using the Mann–Whitney *U* test.

### Elevated plus-maze test

*Mpp6* +/+ and *Mpp6* −/− mice of different age groups were tested (2–3 months: *n* = 6 and 7, respectively; 4–7 months: *n* = 3 and 4, respectively; 18 months: *n* = 3 per group). The elevated plus-maze was constructed using plastic rails (Pla-rail™, Takara-Tomii, Tokyo, Japan) and elevated 40 cm above the floor. Closed arms were enclosed using Truss bridge structures [Pla-rail™; 40 cm (length) × 6 cm (width) × 7 cm (height)]. Mice were placed at the center of the maze, and time spent in the open and closed arms was recorded for 10 min using video tracking. Statistical analysis of the average time spent in different areas (closed arm, center area, and open arm) by *Mpp6* +/+ and *Mpp6* −/− mice at different developmental stages (2–3 months, 4–7 months, and 18 months) was performed using the Mann–Whitney *U* test.

### Locomotor activity (movement) test

To measure locomotor activity, Nano-tag™ devices (Kissei Comtech, Matsumoto, Japan) were implanted subcutaneously in 8-month-old *Mpp6* +/+ and *Mpp6* −/− mice (three per group) under anesthesia. After a 2-week acclimation period, movement was recorded for 7 d. The light cycle was set from 9:00 to 21:00, and the dark cycle from 21:00 to 9:00. Statistical analysis of the average locomotor activity counts per hour in *Mpp6* +/+ and *Mpp6* −/− mice (three per group) was performed using the Mann–Whitney *U* test.

## Results

### Expression of MPP6 and its absence in *Mpp6*-mutant mouse brain

**Fig. 1:**
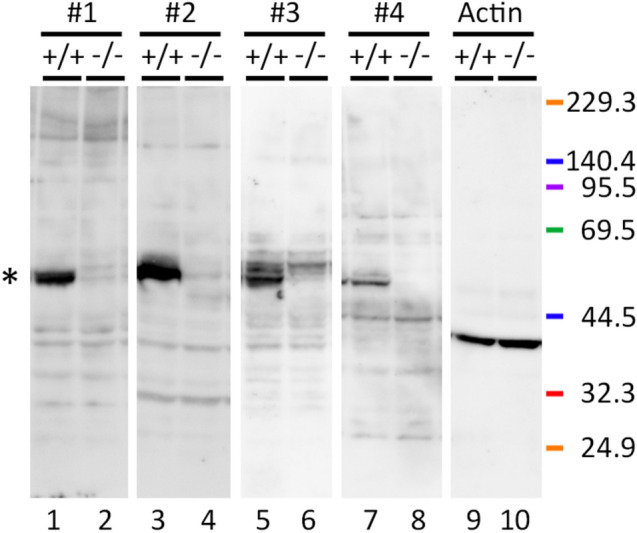
Western blot analysis of MPP6 in the cerebrum of *Mpp6* +/+ and *Mpp6* −/− mice. Four primary anti-MPP6 antibodies (*no. 1 (lanes 1, 2), no. 2 (lanes 3, 4), no. 3 (lanes 5, 6), and no. 4 (lanes 7, 8)*) targeting different domains of the MPP6 protein were used. Detailed antibody information is provided in the *Materials and methods* section

First, MPP6 protein expression in the mouse cerebrum was evaluated by western blot. As shown in Fig. 1, the 55-kDa bands corresponding to the molecular size of MPP6 were clearly detected in *Mpp6* +/+ mice. However, these 55-kDa bands disappeared when using all four anti-MPP6 Abs, each targeting different domains of MPP6, in the *Mpp6*-mutant mouse brain. The *Mpp6* −/− mouse can therefore be considered a MPP6 protein-deficient mouse.

### Expression of MPP6 and related proteins in the cerebral synaptic membranes

**Fig. 2:**
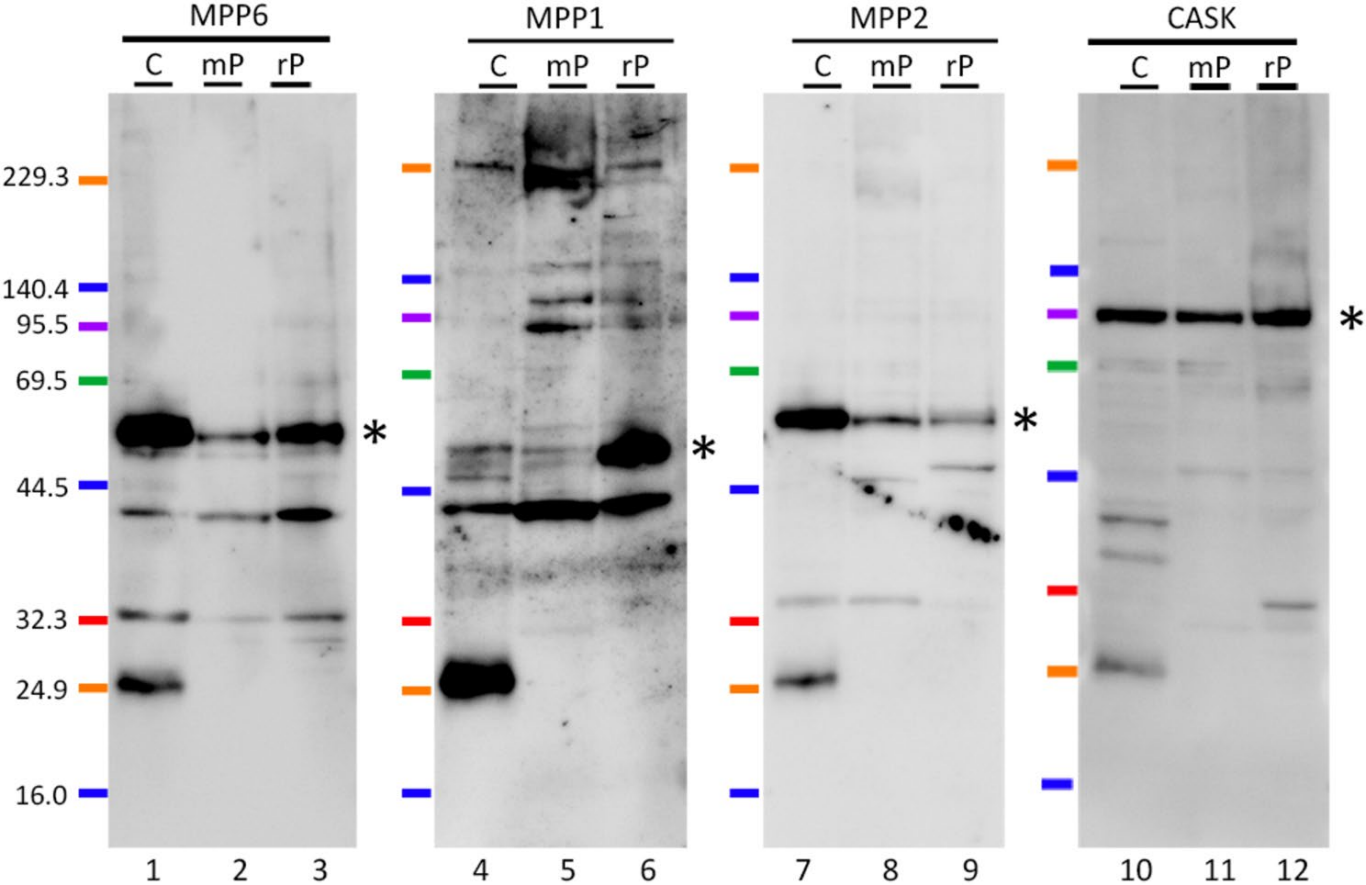
Western blot analysis of MPP family proteins in the synaptic fraction. MPP6 (*lanes 1–3*), MPP1 (*lanes 4–6*), MPP2 (*lanes 7–9*), and CASK (*lanes 10–12*) were analyzed in mouse cerebral lysate (*C; lanes 1, 4, 7, and 10*) and in the synaptic plasma membrane fraction from the mouse (*mP; lanes 2, 5, 8, and 11*) and rat (*rP; lanes 3, 6, 9, and 12*) cerebrum. *Asterisks* on the right side of *lanes 3, 6, 9, and 12* indicate the predicted molecular weights of the MPP6, MPP1, MPP2, and CASK proteins, respectively

To further investigate, the synaptic plasma membrane fraction, including the postsynaptic density (PSD) structure, was obtained by ultracentrifugation of mouse and rat cerebrum. Immunoblot analyses were then performed to detect MPP6 family proteins. As shown in Fig. 2, MPP6, along with other MPP family members MPP1 and MPP2, as well as the PSD scaffolding protein CASK, was detected in both cerebral lysates and synaptic fractions of mouse and rat brains.

**Fig. 3:**
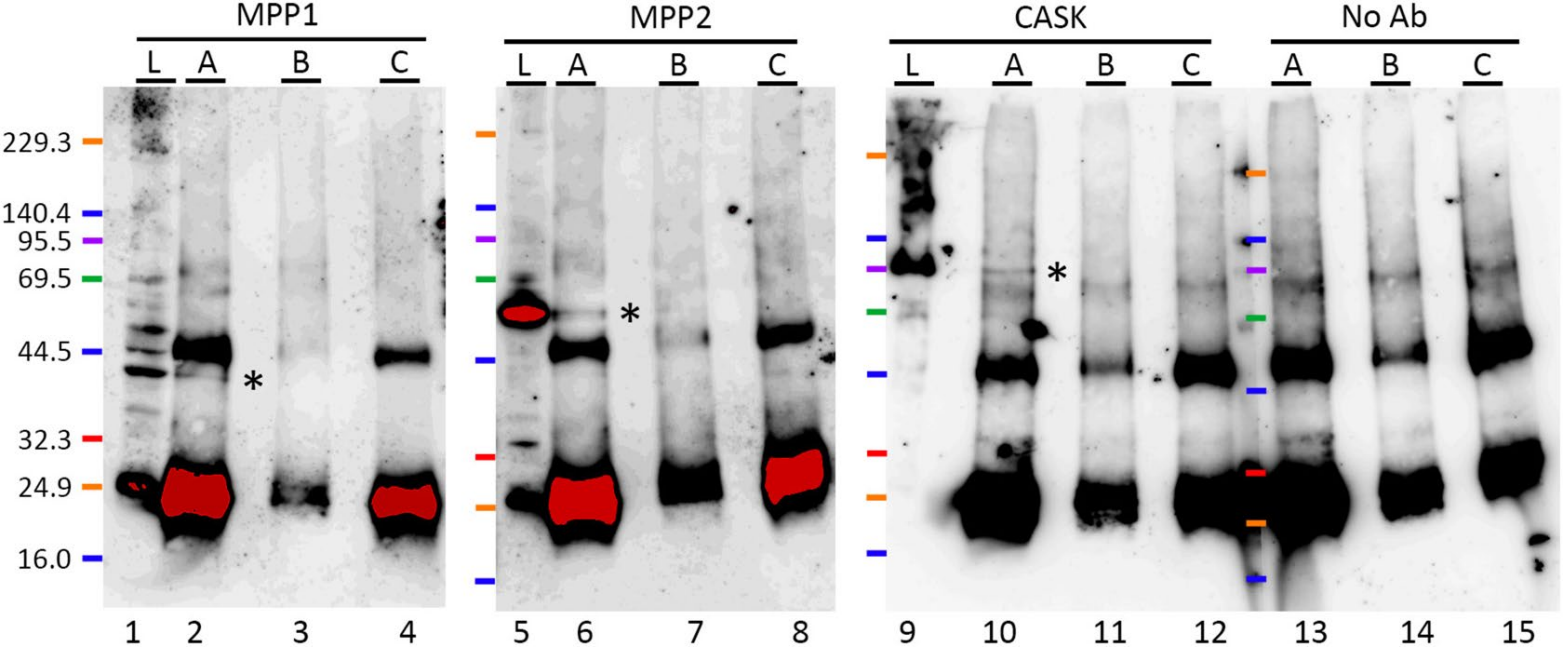
Immunoprecipitation analysis of MPP6 in the cerebrum. Western blot detection using anti-MPP1 (*lanes 1–4*), MPP2 (*lanes 5–8*), CASK (*lanes 9–12*), and no primary antibody (*lanes 13–15*) following immunoprecipitation of cerebral lysates with anti-MPP6 antibody. Sample *A* (*lanes 2, 6, 10, and 13*): cerebral lysate immunoprecipitated with anti-MPP6 antibody, followed by protein G-sepharose collection. Sample *B* (*lanes 3, 7, 11, and 14*): cerebral lysate processed without anti-MPP6 antibody, followed by protein G-sepharose collection. Sample *C* (*lanes 4, 8, 12, and 15*): anti-MPP6 antibody alone, collected with protein G-sepharose. Sample *L* (*lanes 1, 5, and 9*): cerebral lysate. *M*: molecular marker. *Asterisks* on the right side of *lanes 2, 6, and 10* indicate bands corresponding to MPP1, MPP2, and CASK. The exposure time for chemiluminescence detection was extended to ensure the detection of faint signals, leading to red-saturated areas

To examine whether MPP6 interacts with other MPP family proteins in the cerebrum, an immunoprecipitation study was conducted. As shown in Fig. 3, bands corresponding to the predicted molecular weights of MPP1, MPP2, and CASK appeared in the MPP6-immunoprecipitated lysate, indicating the formation of a molecular complex involving MPP6 and these proteins.

**Fig. 4:**
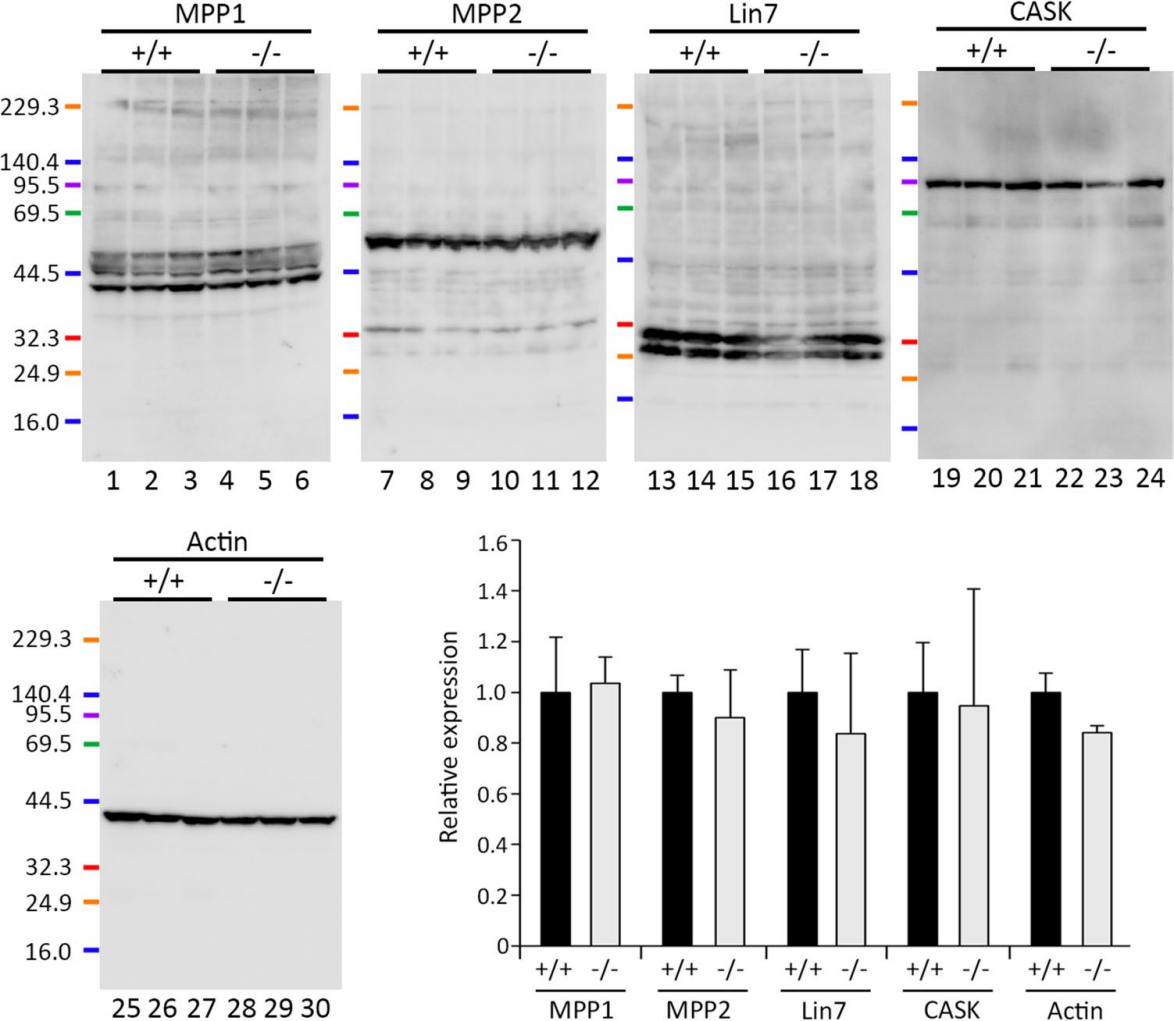
Western blot analysis of MPP1 (*lanes 1–6*), MPP2 (*lanes 7–12*), Lin7 (*lanes 13–18*), CASK (*lanes 19–24*), and actin (*lanes 25–30*) in the cerebrum of *Mpp6* +/+ and *Mpp6* −/− mice. Samples were obtained from three different *Mpp6* +/+ and *Mpp6* −/− mice, and their protein concentrations were adjusted as described in the *Materials and methods* section. Graph showing the relative protein expression ratios of MPP1, MPP2, Lin7, CASK, and actin in *Mpp6 −/− *mice compared with *Mpp6* +/+ mice. Detailed statistical procedures are described in the *Materials and Methods* section

Next, to determine whether MPP6 influences the expression levels of MPP family proteins, western blot analysis was performed to compare the expression of MPP1, MPP2, Lin7, and CASK between *Mpp6* +/+ and *Mpp6* −/− cerebral lysates. Lin7 is a scaffolding protein containing an L27 domain that interacts with MPP6, and its expression and localization were previously reported to be disrupted in Schwann cells under MPP6 deficiency in the peripheral nervous system (Saitoh et al. 2019). As shown by the actual western blot bands and the statistical summary graph in Fig. 4, there was no significant relative expression of MPP1, MPP2, Lin7, and CASK proteins in the cerebrum of *Mpp6* −/− mice, suggesting that MPP6 plays little role in the transport and/or retention of these proteins in the central nervous system.

### Immunolocalization of MPP6 in mouse cerebrum via light microscopy

**Fig. 5:**
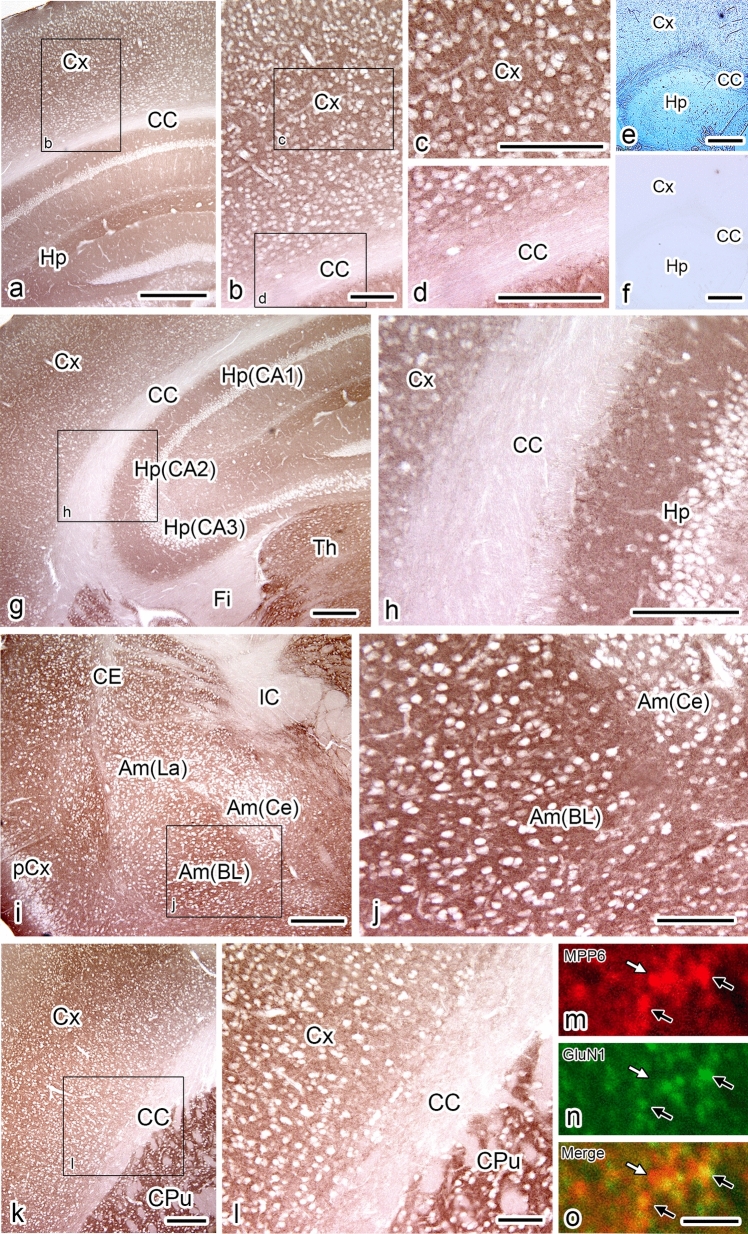
Immunolocalization of MPP6 in the mouse cerebrum using light microscopy. **a**–**l**: floating sections of the cerebrum, including the cerebral cortex (*Cx*: **a**–**c**, **e**–**h**), hippocampus (*Hp*: **a**, **b**, **e**–**h**), amygdala (*Am*: **i,** **j**), and corpus putamen (*CPu*: **k**, **l**). Approximate coronal Allen Atlas levels 70 (**a**–**h**), 62 (**i**, **j**), and 55 (**k**, **l**) immunostained for MPP6. **b**, **c**, **d**, **h**, **j**, and **l**: higher magnifications of the areas shown in **a**, **b**, **g**, **i**, and **k**, respectively. **e, f:** Control immunostain, without the primary antibody [***f:*** differential interference contrast (DIC) image]. *Am (BL, Ce, and La)* basolateral, central, and lateral amygdaloid nucleus, *CA* cornu Ammonis, *CC* corpus callosum, *Fi* fimbria hippocampi, *pCx* piriform cortex. **m**–**o**: confocal laser-scanning microscopic images of MPP6 (red in **m**, **o**) and GluN1 (green in **n**, **o**) in *Cx*. **o** is merged image of **m** and **n**. The white arrow indicates a red-stained area without green staining, whereas the black arrows indicate areas stained with both red and green. *Scale bars*: **a**, **e**, and **f** 500 μm; **g**, **i**, and **k** 200 μm; **b**, **c**, **d**, **h**, **j**, and **l** 100 μm; and **m**–**o** 2 μm

The localization of MPP6 was evaluated using immunohistochemistry. For light microscopic immunohistochemistry on floating sections of mouse cerebrum using an anti-MPP6 antibody, antigen retrieval was enhanced by heat treatment with microwave irradiation in ImmunoSaver™ solution (data not shown). As shown in Fig. 5, MPP6 was immunolocalized in a dot-like pattern in the gray matter across broad areas of the mouse brain, including the cerebral cortex (Fig. 5a–c, g–i, k, l), hippocampus (Fig. 5a, g, h), amygdala (Fig. 5i, j), corpus putamen (Fig. 5k, l), and cerebellar cortex (data not shown). Staining was minimal in white matter, such as the corpus callosum, fimbria hippocampi, and internal capsule, suggesting that MPP6 localization is associated with neurons. In addition, double immunostaining of MPP6 and the glutamate receptor, GluN1, in the cerebral cortex, followed by confocal laser scanning microscopy, revealed the localization of MPP6 in excitatory synapses (Fig. 5m–o). The significance of MPP6-immunostained regions that do not overlap with GluN1 will be discussed in the *Discussion* section.

### Ultrastructural immunolocalization of MPP6 in mouse cerebrum via immunoelectron microscopy

**Fig. 6:**
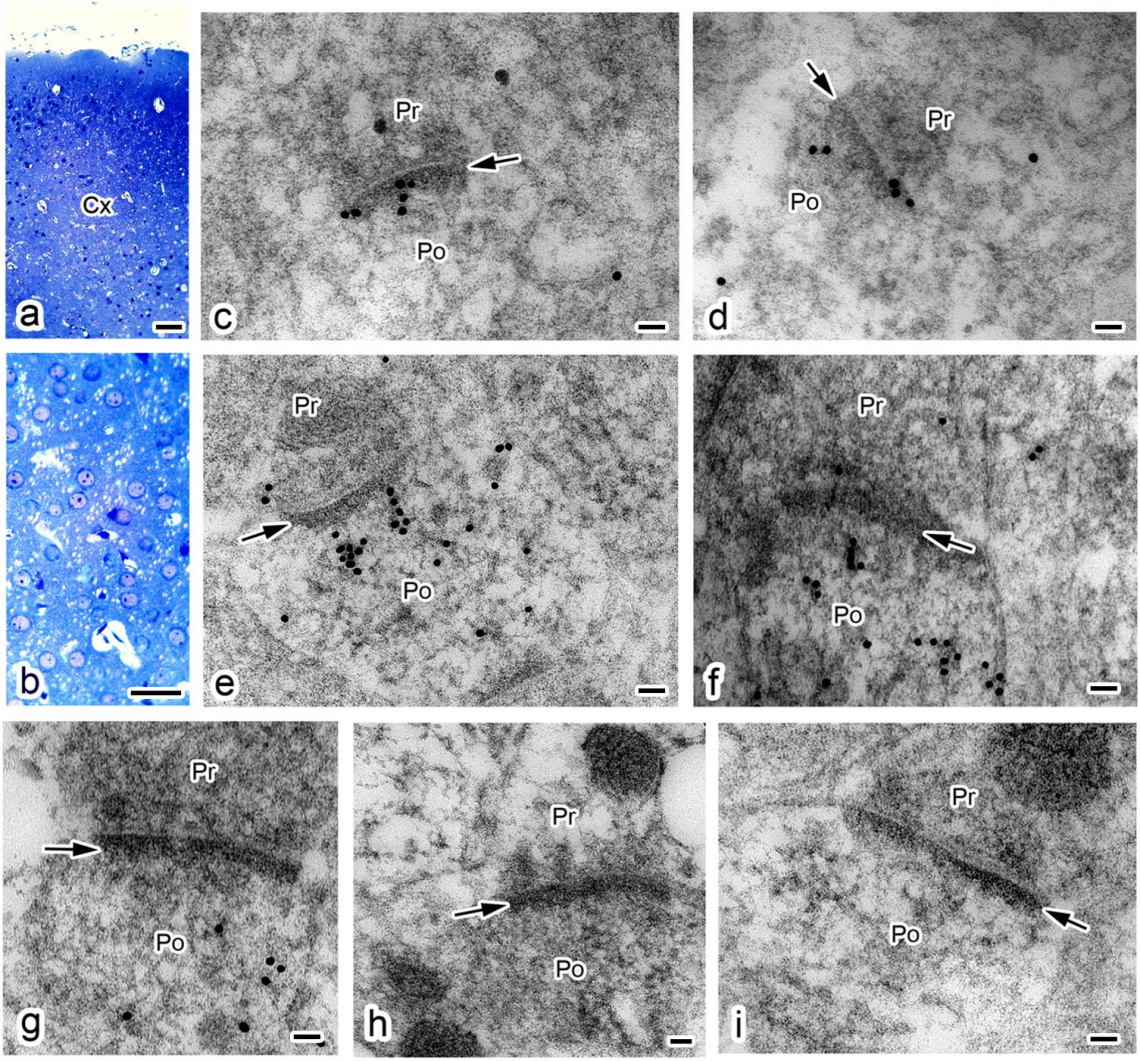
Ultrastructural immunolocalization of MPP6 in the mouse cerebral cortex using post-embedding immunoelectron microscopy. (**a**, **b**) Light microscopic images of thick sections stained with toluidine blue. (**b**) Higher magnification of a region in **a**. (**c**–**i**) Transmission electron micrographs showing 18 nm gold particles conjugated to secondary antibodies localized on and near the postsynaptic densities (*arrows*) following treatment with anti-MPP6 antibody. (**h**, **i**) Control immunostain without primary anti-MPP6 antibody. *Pr* presynaptic site, *Po* postsynaptic site. *Scale bars*: **a** 100 μm; **b** 50 μm; and **c**–**i** 50 nm

The ultrastructural localization of MPP6 in the mouse cerebrum was examined using post-embedding immunoelectron microscopy. Ultrathin sections were obtained from cortical layers II/III–V, as these layers contain relatively well-organized pyramidal cells. Heat treatment by immersing ultrathin sections in ImmunoSaver™ solution at 95 °C was also effective for antigen retrieval, as observed in floating sections for light microscopy. In this study, we focused on type I synapses because mushroom-shaped synapses were clearly identifiable after mild fixation for immunostaining. As shown in Fig. 6, gold particles conjugated to the secondary antibody were observed on and beneath the PSDs in type I synapses. Compared with postsynaptic sites marked by PSDs, fewer gold particles were detected in presynaptic sites, indicating that MPP6 is primarily localized in postsynaptic regions.

### Ultrastructural analysis of synapses in *Mpp6* +/+ and *Mpp6* −/− mouse cerebrum

**Fig. 7:**
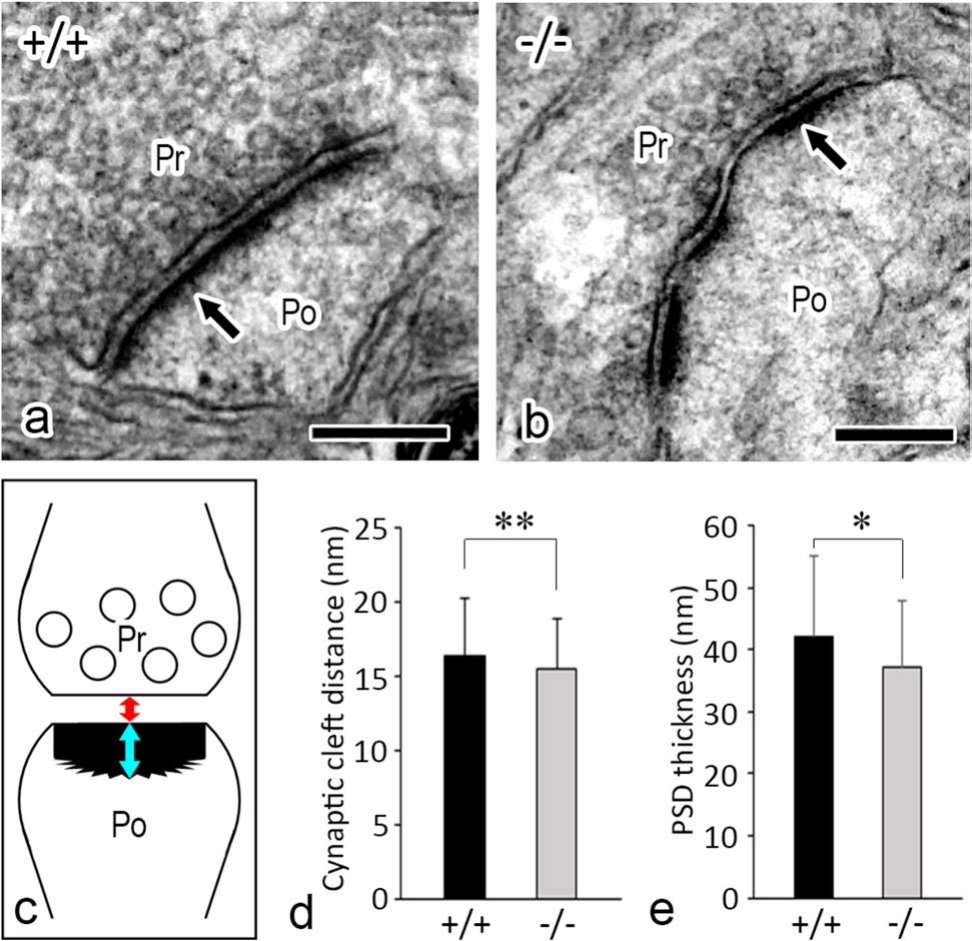
Ultrastructural analysis of synapses in the cerebral cortex of *Mpp6* +/+ and *Mpp6* −/− mice using transmission electron microscopy. (**a**, **b**) Representative electron micrographs of synapses in *Mpp6* +/+ (**a**) and *Mpp6* −/− (**b**) mouse cerebral cortex. *Arrows* indicate the postsynaptic density (PSD). *Pr* presynaptic site, *Po* postsynaptic site. (**c**) Schematic representation of a synapse illustrating the synaptic cleft distance *(red double-headed arrow)* and PSD thickness *(blue double-headed arrow)*. (**d**) Graph depicting the synaptic cleft distance between presynaptic and postsynaptic membranes (*n* = 280 for both *Mpp6* +/+ and *Mpp6* −/− mice). (**e**) Graph showing PSD thickness (*n* = 375 for both *Mpp6* +/+ and *Mpp6* −/− mice). *p < 0.05, **p < 0.01. *Scale bars:* **a**, **b** 200 nm

The ultrastructure of excitatory synapses was compared between *Mpp6* +/+ and *Mpp6* −/− cerebral cortices using a transmission electron microscope. Ultrathin sections were obtained from cortical layers II/III–V. Measurements of synaptic cleft distance and PSD thickness in mushroom-shaped type I synapses are presented in Fig. 7. Statistically, *Mpp6* −/− mice exhibited thinner synaptic clefts and PSDs compared with *Mpp6* +/+ mice, indicating slight morphological alterations in the synapses of *Mpp6* −/− mice.

### Elevated plus-maze test for *Mpp6* +/+ and *Mpp6* −/− mice

**Fig. 8:**
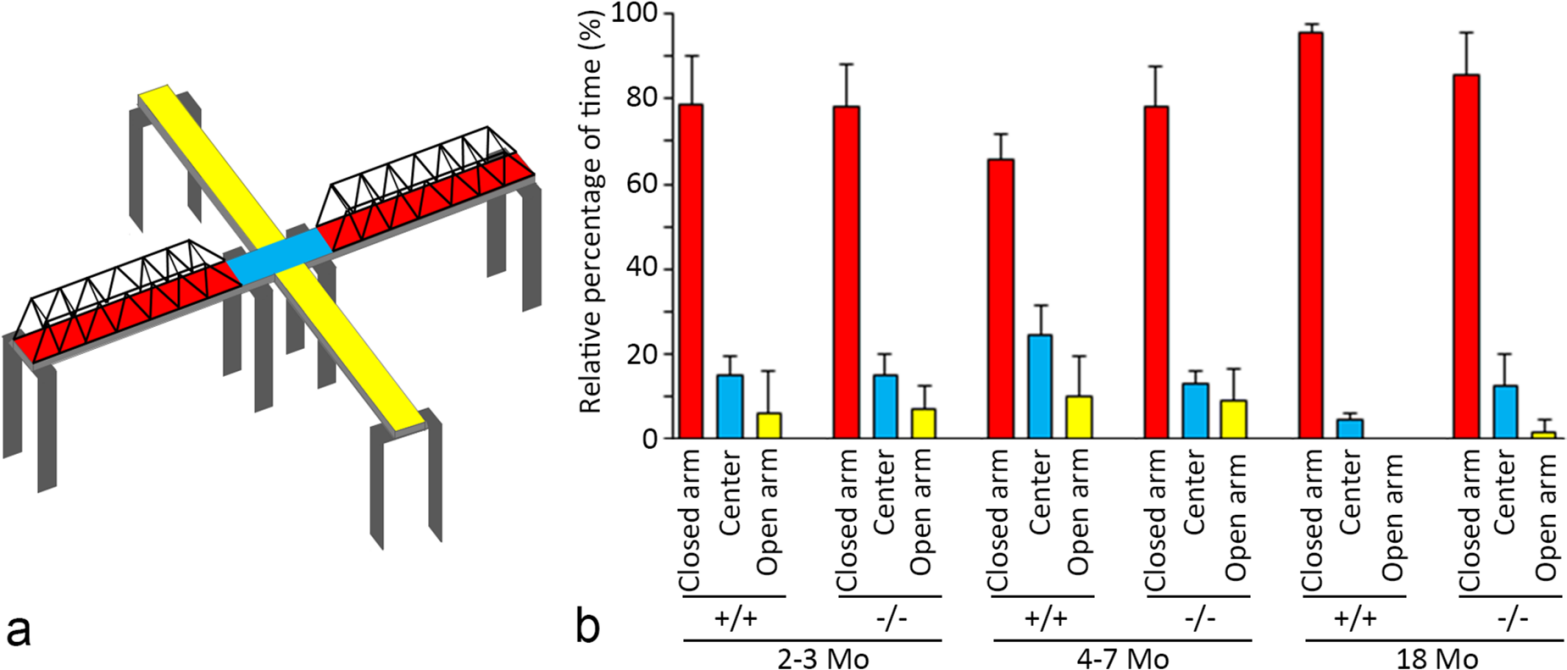
Elevated plus-maze test. (**a**) Schematic diagram of the apparatus used for the elevated plus-maze test. Detailed construction methods are described in the *Materials and methods* section. The test began with the mouse placed in the center area. The total time (10 min) spent in the center (*blue* area), closed arm (inside the truss bridge; *red* area), and open arm (*yellow* area) was measured for each mouse. (**b**) Graph showing the relative percentage of time spent in different areas (closed arm: *red*; center: *blue*; and open arm: *yellow*) for *Mpp6* +/+ and *Mpp6* −/− mice at different developmental stages: 2–3 months *(2–3 mo.)*, 4–7 months *(4–7 mo.)*, and 18 months *(18 mo.)*

*MPP6* has been identified through GWAS as a gene associated with certain human mental disorders, including schizophrenia and bipolar disorder (Ripke et al. 2014). To investigate whether MPP6 deficiency affects anxiety-related behavior, an elevated plus-maze test was conducted. In this experiment, a Truss bridge was originally introduced as a safety zone, analogous to the closed arm, and its suitability for the elevated plus-maze test was initially evaluated. As shown in Fig. 8, *Mpp6* +/+ mice statistically maintained their safety in the closed arms compared with the open arms (*p* < 0.0001). However, comparisons between *Mpp6* +/+ and *Mpp6* −/− mice across three different developmental age groups (with precise sample sizes described in the *Materials and methods*) revealed no statistically significant differences in the time spent in the open and closed arms. Interestingly, one 2- to 3-month-old *Mpp6* −/− mouse exhibited unusual behavior by climbing over the top struts of the Truss bridge for 50 s.

### Locomotor activity test for *Mpp6* +/+ and *Mpp6 *−/− mice

**Fig. 9:**
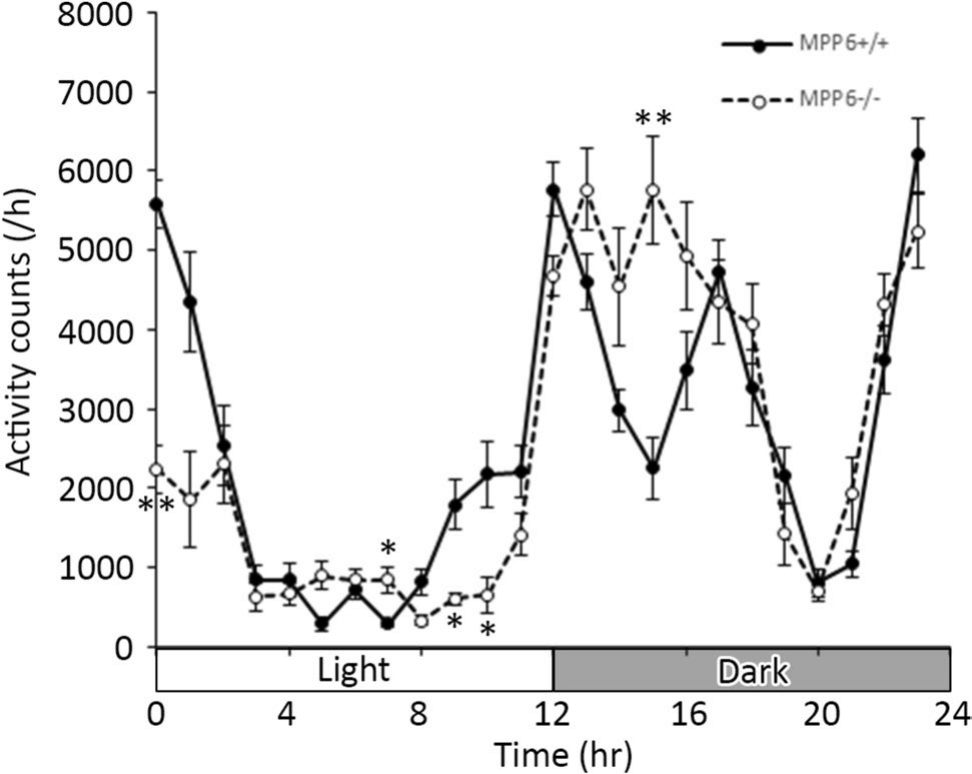
Locomotor activity test. Detailed methods for measuring activity are described in the *Materials and methods* section. The graph shows the average locomotor activity counts per hour for *Mpp6* +/+ and *Mpp6* −/− mice (three mice per group). *p < 0.05, **p < 0.01, compared at the same times across each hour

Since *MPP6* has been reported as a gene associated with sleep regulation in humans (Khoury et al. 2021), daily locomotor activity was assessed. As shown in Fig. 9, *Mpp6* −/− mice exhibited reduced activity during the daytime and increased activity at night compared with *Mpp6* +/+ mice. This suggests that MPP6 may play a role in sleep–wake regulation.

## Discussion

In this study, we clarified that MPP6 is one of the synaptic proteins, particularly in the postsynaptic region, and forms molecular complexes with other MPP family proteins in the CNS. Except for MPP1, MPP family proteins possess two L27 domains, which enable them to interact with each other (Chytla et al. 2020). Our present study (Fig. 3) demonstrated that immunoprecipitated cerebral lysate using an anti-MPP6 Ab contained MPP1, MPP2, and CASK. Previous studies have reported that MPP6 and MPP2 exhibit high molecular similarity (Velthuis et al. 2007), and immunolocalization of MPP2 overlaps with that of MPP6 (data not shown). MPP1 was previously identified as a PSD protein (Zhang et al. 2005). In a MPP1-null mouse model, ectrodactyly, including facial and eye abnormalities, as well as hydrocephaly, was reported (Fritz et al. 2019). Although MPP1 lacks the L27 domain, our present study found that MPP6 forms a molecular complex with MPP1 in cerebral lysate. Since the GK domain has also been suggested as an interaction site (Ye et al. 2018), further examination of the association between MPP1 and MPP6 is required. Regarding CASK, it has been reported to localize to both presynaptic and postsynaptic sites (Fallon et al. 2002). Our ultrastructural immunohistochemical study (Fig. 6) revealed that MPP6 is primarily localized in the postsynaptic region. Given that CASK shares a similar molecular structure with MPP6 in the PDZ, SH3, and GK domains (Velthuis et al. 2007), an important next step is to elucidate how MPP6 and CASK interact in synapses. In this study, we demonstrated that MPP6 localization partially overlaps with GluN1-immunostained excitatory synapses (Figs. 5 and 6). However, we also observed MPP6-immunostained areas that do not overlap with GluN1-immunostained areas (Fig. 5). Given that the colocalization of the γ-aminobutyric acid (GABA) receptor with MPP2, a member of the MPP family, has been reported in neuronal cultures (Schmerl et al. 2022) and cerebellar tissue (Terada et al. 2023), it remains an open question whether MPP6 is also present in type II synapses, including inhibitory synapses. This warrants further investigation.

Recent GWAS have demonstrated a relationship between *MPP6* and various psychiatric disorders, including schizophrenia (Fritz et al. 2019; Ganapathiraju et al. 2016; Greenwood et al. 2019; Le Hellard et al. 2017; Lin et al. 2016), bipolar disorder (Mullins et al. 2021), and autism spectrum disorder (Chang et al. 2015; Guo et al. 2019; Le Hellard et al. 2017; Li et al. 2014). *MPP6* has also been implicated in at least two psychiatric disorders among anorexia nervosa, attention-deficit/hyperactivity disorder, major depression, obsessive–compulsive disorder, schizophrenia, and Tourette syndrome (Lee et al. 2019). Interestingly, *MPP6* was identified as 1 of 57 hard sweep genes following the initial migration of anatomically modern humans out of Africa (Tobler et al. 2023). In mice, the *Mpp6* gene was associated with altered gene expression profiles in BTBRTF/ArtRbrc mice, which exhibit low sociability (Mizuno et al. 2020). Moreover, CASK has been linked to psychiatric behavior in both humans and mouse models (Hsueh 2009; Huang and Hsueh 2017; Mori et al. 2019). In the present study (Fig. 7), ultrastructural analysis revealed that PSD thickness was slightly reduced in *Mpp6* −/− mice compared with *Mpp6* +/+ mice. Among the proteins associated with psychiatric disorders that contribute to the formation of postsynaptic sites, including the PSD, an intriguing question is how morphological changes occur in their absence. For instance, a reduction in PSD thickness has been reported in the absence of SH3 and multiple ankyrin repeat domains 3b (Shank3b), a postsynaptic scaffold protein linked to social deficits (Wang et al. 2019). Our present study included behavioral analysis using the elevated plus-maze test (Fig. 8). Although one *Mpp6* −/− mice exhibited markedly unusual exploratory behavior, significant differences in anxiety levels compared with *Mpp6* +/+ mice were not observed. However, as *Mpp6* +/+ mice never exhibited such behavior, it remains unclear whether this anomaly is directly related to MPP6 deficiency. Regarding anxiety behavior, a limitation of the present study is that we only performed the elevated plus maze test. Additional behavioral experiments, such as novel object recognition, fear conditioning, and conditioned place aversion (Otsu et al. 2019), would be valuable for future studies.

Conversely, a GWAS study on sleep disorders identified novel genome-wide loci on human chromosome 7, including *MPP6*. Disruption of an ortholog of Mpp6 in *Drosophila melanogaster* led to altered sleep patterns (Khoury et al. 2021). In our present study, we observed differences in activity during wake and sleep periods between *Mpp6* +/+ and *Mpp6* −/− mice (Fig. 9). Interestingly, *Mpp6* +/+ mice exhibited increased activity at night. Considering synaptic proteins related to the MPP family, SAP90-associated proteins (SAPAP)/discs large-associated proteins (DLGAP) have been identified as GK domain-binding partners of MPP family members and related membrane-associated guanylate kinase (MAGUK) proteins (Rasmussen et al. 2017). Notably, SAPAP4-deficient mice have been reported to exhibit hyperactivity during wake periods, serving as a model of cognitive impairment and autism-like behavior (Schob et al. 2019). Furthermore, sleep–wake states are believed to be influenced by reversible protein phosphorylation involving kinases such as calcium–calmodulin (CaM)-dependent protein kinase II (CaMKII) and protein kinase A (PKA) in synapses—a concept known as the “phosphorylation hypothesis of sleep” (Ode and Ueda 2020; Wang et al. 2024). Therefore, further studies are necessary to determine how MPP6 contributes to synaptic signaling and neuronal networks related to behavior.

With MPP families, other MAGUK proteins, such as the Dlg family (Dlg1–4), may also form oligomers through their PDZ–SH3–GK tandem domains (Ye et al. 2018). In synapses, Dlg family proteins play key roles in postsynaptic density formation and signal transduction (Velthuis et al. 2007; Zhu et al. 2016). Dysfunction of the Dlg family has been implicated in several mental disorders: Dlg4 (also known as PSD95 or SAP90) (Coley and Gao 2018; Funk et al. 2017; Gao and Mack 2021; Grant 2016; Levy et al. 2022; Nagura et al. 2012; Nithianantharajah et al. 2013; Xing et al. 2016), Dlg1 (also known as SAP97) (Gupta et al. 2018; Kay et al. 2022; Uezato et al. 2017), and Dlg2 (also known as PSD93 or Chapsin110) (Winkler et al. 2018; Yoo et al. 2020). While the Dlg family proteins appear to function redundantly in synaptogenesis and synaptic functions by interacting with neurotransmitter receptors and adhesion molecules (Oliva et al. 2012), an intriguing finding is that the nano-organization of Dlg3 and Dlg4 differs within single synapses (Metzbower et al. 2024). Since the molecular evolution of vertebrate behaviors appears to be linked to the diversity of MAGUK proteins, including both Dlg and MPP families (Grant 2016), further investigations into large molecular complexes within individual synapses may provide deeper insights into neurological and psychological functions.

## Data Availability

No datasets were generated or analyzed during the current study.
